# Accuracy and reliability of a continuous glucose monitoring system with a focus on hypoglycaemia

**DOI:** 10.1111/dom.70408

**Published:** 2025-12-25

**Authors:** Lukas van Baal, Lorenz Raess, Annie Mathew, Harald Lahner, Nicole Unger, Dagmar Fuhrer

**Affiliations:** ^1^ Department of Endocrinology, Diabetes and Metabolism, University Hospital Essen University Duisburg‐Essen Essen Germany

**Keywords:** accuracy, continuous glucose monitoring (CGM), hospital, hypoglycaemia, inpatient, reliability

## Abstract

**Aims:**

Glucose monitoring is increasingly based on continuous glucose monitoring (CGM) systems. However, data on the accuracy of CGM within the hypoglycaemic range is sparse. This study investigated CGM accuracy within the hypoglycaemic range in three different clinical settings of hypoglycaemia.

**Materials and Methods:**

Ninety‐two people with various causes of hypoglycaemia were analysed: (i) people during insulin tolerance testing (ITT) (*n* = 63); (ii) people with insulinoma (*n* = 16); and (iii) people with diabetes receiving subcutaneous insulin therapy (*n* = 13). CGM accuracy was evaluated for subgroups and different glucose rates of change (RoC) using mean absolute relative difference (MARD), percentage of glucose values within ±20 mg/dL of point‐of‐care glucose (%20/20), diabetes technology society error grid and Bland–Altman analysis (BAA).

**Results:**

Four hundred sixty‐four CGM/POC glucose pairs were obtained (39.7% level 1, 60.3% level 2 hypoglycaemia). CGM accuracy decreased from people with diabetes receiving subcutaneous insulin therapy (MARD: 13.9%; %20/20: 81.5%) to those with ITT (MARD: 50.8%; *p* < 0.01, %20/20: 67.5%; p .02). CGM accuracy significantly decreased with higher RoC. Proportion of CGM values with moderate risk in failing to detect potentially dangerous hypoglycaemia increased from people with diabetes (1.5%) and insulinoma (1.2%) to people with ITT (14.2%, *p* < 0.01). BAA revealed a significantly increasing bias of −3.3 + 10.7 mg/dL in people with diabetes receiving insulin therapy to −15.2 ± 13.6 mg/dL in people with ITT (*p* < 0.01).

**Conclusion:**

CGM accuracy can vary in different clinical hypoglycaemia scenarios. While it remains acceptable in people with diabetes receiving subcutaneous insulin therapy and people with insulinoma, it appears to be inaccurate for glucose monitoring during ITT.

## 1 INTRODUCTION

Hypoglycaemia has been reported in up to 22.0% of hospitalised people and is strongly associated with the prevalence of diabetes.^1^, ^2^, ^3^ The standard treatment for achieving glucose control in an inpatient setting is insulin, which aims for “near normoglycemia.” However, insulin confers a threefold higher risk of hypoglycaemia compared to conventional diabetes therapy without use of insulin, finally causing life‐threatening events such as seizures and cardiovascular events, as well as emotional distress for people.^4^, ^5^ Consequently, insulin has been classified as a “high‐alert” medication that can cause fatal harm when used incorrectly, necessitating close glucose monitoring.^6^ The standard of care for in‐hospital glucose monitoring is multiple daily measurements of capillary blood glucose using point‐of‐care blood glucose monitoring (POC‐G), resulting in an average of four to six glucose measurements per day. However, this method is insufficient for detecting hypoglycaemia, especially in people with impaired hypoglycaemia awareness.^7^, ^8^, ^9^, ^10^ Furthermore, this procedure is inconvenient for people and time‐consuming for nursing staff. Therefore, continuous glucose monitoring (CGM) systems may be an alternative. Large, randomised, controlled trials have demonstrated that CGM use significantly reduces the incidence and duration of hypoglycaemia in outpatient settings.^11^, ^12^, ^13^ In contrast, CGM use in inpatients is still considered investigational.^14^, ^15^, ^16^ Recognising preliminary data and technical advantages, some guidelines have started to recommend CGM use for inpatients at high risk for hypoglycaemia.^17^, ^18^ However, data on the reliability and accuracy of CGM within the hypoglycaemic range are lacking. Here, we analysed CGM accuracy in three different scenarios of hypoglycaemia in controlled inpatient settings: (i) people with insulin tolerance testing (ITT), (ii) people with insulinoma, and (iii) people with diabetes receiving subcutaneous insulin therapy.

## 2 MATERIALS AND METHODS

### 2.1 General study design

This single‐centre, prospective study was conducted at the Department of Endocrinology, Diabetology, and Metabolism at the University Hospital Essen. The accuracy and reliability of CGM within the hypoglycaemic range were investigated in three different scenarios in people admitted to our department from March 1, 2022, to August 31, 2023. (1) People receiving intravenous insulin aiming for a glucose value of <40 mg/dL in ITT for the investigation of corticotropin deficiency. (2) People with insulinoma; and (3) People with diabetes receiving subcutaneous insulin therapy and requiring therapy adjustments. As part of the university hospital's quality improvement project, SmartDiabetesCare (QiP SDC), all included people received a CGM (FreeStyle Libre 3, Abbott, Wiesbaden, Germany) upon admission, applied by a member of the diabetes team. The CGM measured subcutaneous interstitial fluid glucose concentrations continuously. The people also received a smartphone (iPhone SE, Apple, USA), which received data from the CGM via an application (FreeStyle LibreLink). The sensor remained in place until the day of discharge or for a maximum of 14 days. If the length of the hospital stay exceeded the wear time, the first sensor was removed and a second sensor was inserted. Additionally, the healthcare teams were instructed to remove the sensor in case of an MRI examination or a local skin reaction. However, this was not necessary in any participant.

### 2.2 Procedure of insulin tolerance testing

ITT was performed using a validated standard operating procedure of the department. Blood glucose was measured with a POC‐G 15 min before the start of the test, after which an insulin bolus adjusted by HbA1c and body weight was calculated by a senior physician and administered 15 min later. Blood glucose values were then measured at 15, 30, 45, 60, 90 and 120 min. The test ended after 120 min. A nurse monitored vital parameters and consciousness closely throughout the test. In case of loss of consciousness or seizure, an intravenous bolus of 60 mL of G40% was administered immediately. Individuals with known coronary artery calcifications, a history of myocardial infarction, or a history of seizures were not permitted to undergo the test. Glucose values were obtained from plasma (Plasma‐G), point‐of‐care glucose (POC‐G), and continuous glucose monitoring (CGM‐G) at each time. The glucose data were documented in the person's electronic health record (EHR), resulting in at least seven CGM/POC‐G and CGM/Plasma‐G pairs per person and test. Plasma‐G was immediately brought to our laboratory division and measured using the hexokinase method. Glucose in the sample is phosphorylated with ATP in the presence of hexokinase. The resulting glucose‐6‐phosphate is oxidised by glucose‐6‐phosphate dehydrogenase, which reduces NAD to NADH. Photometry was performed at 340 nm after deducting the absorbance of the buffer, ATP, and NAD (Atellica CH, Siemens Healthineers, Forchheim, Germany). POC‐G was measured immediately by our nurses beside the person using the StatStrip glucose metre (Nova Biomedical, Mörfelden‐Walldorf, Germany). The coefficient for the slope of the laboratory glucose measurement system (Yellow Springs Instrument) versus the StatStrip glucose meter was 1.023 (*r* = 0.989), and all StatStrip glucose metre readings were within the A zone of the Clark error grid analysis (CEG).^19^

### 2.3 Procedure for people with insulinoma

For people with insulinoma, the nursing staff was asked to document glucose values obtained by CGM once per hour. For people with diabetes receiving subcutaneous insulin therapy, CGM‐G was documented at least four times per day (before meals and once at night). POC‐G was measured at least every 6 h (twice per early and midday shift and once per night shift) with a maximum time lag of less than 2 min from the CGM measurement using a StatStrip glucose meter. Glucose data were documented alongside corresponding CGM‐G data in the person's EHR.

### 2.4 Additional information

Clinical and anthropometric data were extracted from the EHR. The EHRs of the study participants were also checked for substances that are known to interfere with CGM glucose measurements (acetaminophen >4 g/d, acetylsalicylic acid >100 mg/d, ascorbic acid >500 mg/d, or tetracycline).^20^

Informed consent was obtained from all participants included in the study. This study was performed in accordance with the principles of the Declaration of Helsinki. The study was approved by the ethics committee of the University Hospital Essen (20‐9333‐BO).

## 3 STATISTICAL ANALYSIS

To assess CGM accuracy, the following outcome measures were used: (1) the mean absolute relative difference (MARD) between CGM‐G and POC‐G, as well as between CGM‐G and Plasma‐G within the following glucose ranges: <54 mg/dL (level 2 hypoglycaemia), 54–69 mg/dL (level 1 hypoglycaemia), and all hypoglycaemic values, and (2) the number and percentage of glucose values within ±20 mg/dL of POC‐G. The latter is in accordance with the AIDING study, which provides a modified version of the 20/20 agreement rate of the Food and Drug Administration (FDA) criteria for CGM use and is similar to protocols developed by others.^21^, ^22^, ^23^ For clarity, the term “%20/20” will be used throughout the rest of the manuscript. To calculate the MARD, the relative difference (RD) between each CGM glucose measurement result and the point‐of‐care system result was calculated as RD = 100 * (CGM‐G − POC‐G)/POC‐G. Non‐paired glucose values were excluded from the data analysis. Then, the MARD of the CGM/POC‐G pairs was calculated for each person individually and for the respective subgroups as a whole. To assess CGM reliability, Diabetes Technology Society Error Grid (DTS Error Grid) and Bland–Altman analysis (BAA) were performed as previously described.^24^, ^25^

Moreover, the glucose rate of change (RoC) was analysed using the complete CGM traces, with the difference between the documented CGM‐G and the CGM‐G 15 min prior being calculated, and the RoC per minute (mg/dL/min) for these 15 min subsequently determined. Five RoC categories (<−1, −1 to <−0.5, −0.5 to <0.0, ≥0.0 to 0.5 and >0.5 mg/dL/min) were established.

In the ITT setting, accuracy analyses were conducted for paired glucose values (CGM‐G/Plasma‐G and CGM‐G/POC‐G) assessed simultaneously, as well as for paired glucose values that were assessed 15 min apart.

The resulting number of pairs was sufficient to depict medium effect sizes (*f* = 0.25) with good statistical power (0.9).

All calculations were performed using the Statistical Program for Social Sciences (SPSS) version 28 (IBM, New York, USA). The graphs were generated using GraphPad Prism (GraphPad Software, Inc., San Diego, CA). A *p*‐value of less than 0.05 was considered statistically significant.

## 4 RESULTS

### 4.1 People characteristics

A total of 92 people were included in the analysis: 68.5% (63/92) with ITT, 17.4% (16/92) had insulinoma, and 14.1% (13/92) diabetes receiving subcutaneous insulin therapy. The people were 56.5% female, with a mean age of 50.2 ± 15.6 years and a mean BMI of 27.7 ± 8.3 kg/m^2^. Within this cohort, 464 CGM/POC‐G and 295 CGM/plasma‐G measurements within the hypoglycaemic range (minimum: 20 mg/dL; maximum: 69 mg/dL) were obtained (39.7% level 1 hypoglycaemia; 60.3% level 2 hypoglycaemia).

Regarding potentially interfering substances, 13.0% of people were treated with acetylsalicylic acid. However, none received doses greater than 100 mg per day, and 2.2% received acetaminophen, with no person exceeding a daily dose of 4 g. None of the people were treated with ascorbic acid or tetracycline. Table S1 provides detailed demographic information of the population studied.

### 4.2 Accuracy and reliability of CGM

CGM accuracy increased significantly from people with ITT (MARD: 50.8%; %20/20: 67.5%) to people with insulinoma (MARD: 27.2% [*p* < 0.01]; %20/20: 95.1% [*p* < 0.01]; Tables 1 and 2) and people with diabetes receiving subcutaneous insulin therapy (MARD: 13.9% [*p* < 0.01]; %20/20: 81.5% [*p* 0.02]; Tables 1 and 2). Furthermore, during ITT and in people with insulinoma, CGM accuracy was higher during level 1 hypoglycaemia than during level 2 hypoglycaemia (*p* < 0.01 for both; Tables 1 and 2). In people with diabetes receiving subcutaneous insulin therapy, CGM accuracy did not differ between level 1 and level 2 hypoglycaemia. However, this analysis was based on a limited sample of only six values within level 2 hypoglycaemia.

**TABLE 1:** MARD (%) values of different methods of blood glucose measurement.

	MARD CGM‐G to POC‐G (*n*)	MARD CGM‐G to Plasma‐G (*n*)
People with ITT		
Hypoglycaemia overall (< 70 mg/dL)	50.8 (317)	40.6 (295)
Level 1 hypoglycaemia (69–54 mg/dL)	18.4 (93)***	20.0 (78)***
Level 2 hypoglycaemia (<54 mg/dL)	64.3 (224)***	48.0 (217)***
People with insulinoma		
Hypoglycaemia overall (< 70 mg/dL)	27.2 (82)	n.a.
Level 1 hypoglycaemia (69–54 mg/dL)	11.0 (35)***	n.a.
Level 2 hypoglycaemia (<54 mg/dL)	39.3 (47)***	n.a.
People with diabetes receiving subcutaneous insulin therapy		n.a.
Hypoglycaemia overall (< 70 mg/dL)	13.9 (65)	n.a.
Level 1 hypoglycaemia (69–54 mg/dL)	13.2 (59)	n.a.
Level 2 hypoglycaemia (<54 mg/dL)	21.5 (6)	n.a.

Abbreviations: CGM‐G, continuous glucose monitoring glucose value; ITT, insulin hypoglycaemia test; POC‐G, point of care capillary glucose value; Plasma‐G, plasma glucose value.

*** *p* < 0.001 for the difference in MARD between level 1 and 2 hypoglycaemia.

**TABLE 2:** Blood glucose values (%) within ±20 mg/dL regarding different methods of blood glucose measurement.

	%20/20 CGM‐G to POC‐G (*n*)	%20/20 CGM‐G to Plasma‐G (*n*)
People with ITT		
Hypoglycaemia overall (< 70 mg/dL)	67.5 (214/317)	74.5 (211/295)
Level 1 hypoglycaemia (69–54 mg/dL)	87.1 (81/93)***	78.2 (61/78)
Level 2 hypoglycaemia (<54 mg/dL)	59.4 (133/224)***	69.1 (150/217)
People with insulinoma		
Hypoglycaemia overall (< 70 mg/dL)	95.1 (78/82)	n.a.
Level 1 hypoglycaemia (69–54 mg/dL)	97.1 (34/35)	n.a.
Level 2 hypoglycaemia (<54 mg/dL)	93.6 (44/47)	n.a.
People with diabetes receiving subcutaneous insulin therapy		n.a.
Hypoglycaemia overall (< 70 mg/dL)	81.5 (53/65)	n.a.
Level 1 hypoglycaemia (69–54 mg/dL)	84.8 (50/59)	n.a.
Level 2 hypoglycaemia (<54 mg/dL)	50.0 (3/6)	n.a.

Abbreviations: CGM‐G, continuous glucose monitoring glucose value; POC‐G, point of care capillary glucose value; Plasma‐G, plasma glucose value; ITT, insulin hypoglycaemia test.

*** *p* < 0.001 for the difference in proportion of CGM‐G values within ±20 mg/dL of POC‐G value between level 1 and 2 hypoglycaemia.

**FIGURE 1:**
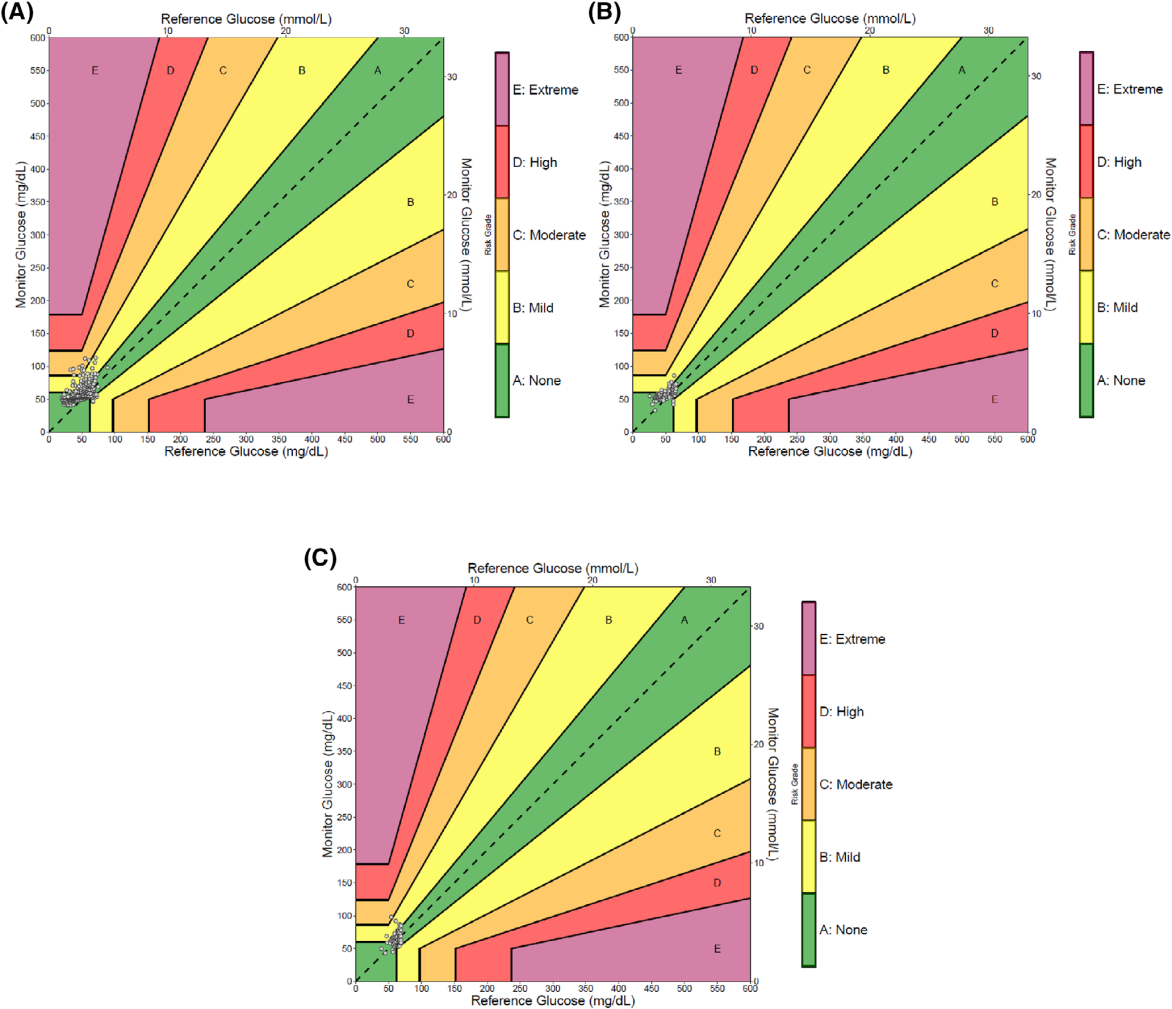
Diabetes technology society error grid CGM‐G in reference to POC‐G. (A) Insulin tolerance testing, (B) people with insulinoma, (C) people with diabetes receiving subcutaneous insulin therapy.

DTS error grid analysis revealed that the proportion of CGM‐G values that presented at least a moderate risk for failing to detect a potentially dangerous hypoglycaemia increased significantly from people with diabetes receiving subcutaneous insulin therapy (1.5%) and people with insulinoma (1.2%) to people with ITT (14.2%; *p* < 0.01; Figure 1).

**FIGURE 2:**
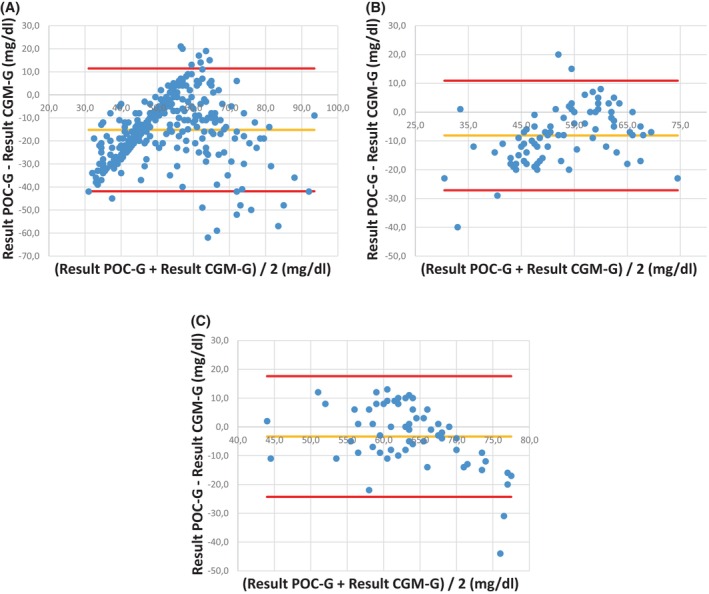
Bland–Altman analysis for CGM‐G in reference to POC‐G in the three scenarios. (A) Insulin tolerance testing, (B) people with insulinoma, (C) people with diabetes receiving subcutaneous insulin therapy. POC‐G, point of care glucose value; CGM‐G, continuous glucose monitoring glucose value.

BAA revealed a negative bias in all three scenarios, with a significantly increasing bias of −3.3 ± 10.7 mg/dL in people with diabetes receiving insulin therapy to −8.1 ± 9.7 mg/dL in people with insulinoma (*p* < 0.01) to finally −15.2 ± 13.6 mg/dL in people with ITT (*p* < 0.01) (Figure 2). The distance between the 95% limits of agreement and the range in which the actual blood glucose would lie with a 95% probability increased marginally from people with insulinoma (−27.1 to 10.9 mg/dL; ±19.0 mg/dL) to people with diabetes receiving subcutaneous insulin therapy (−24.3 to 17.6 mg/dL; ±21.0 mg/dL) and considerably to people with ITT (−41.9 to 11.5 mg/dL; ±26.7 mg/dL) (Figure 2).

A subgroup analysis was performed excluding people taking potentially interfering medications (=exclusion of 71 CGM‐/POC‐G pairs). However, no significant difference was found regarding the aforementioned analyses of CGM accuracy.

### 4.3 Impact of glucose rate of change

RoC was significantly lower in people with diabetes receiving subcutaneous insulin therapy (−0.3 ± 0.5 mg/dL/min) and people with insulinoma (−0.1 ± 0.5 mg/dL/min) than in people with ITT (−0.5 ± 1.1 mg/dL/min; *p* < 0.01). This difference was particularly pronounced at minutes 15 (−1.3 ± 1.2 mg/dL/min; *p* < 0.01) and 30 (−1.3 ± 1.2 mg/dL/min; *p* < 0.01) of the ITT. Consequently, the proportion of CGM‐G values in the range of a RoC <−1 mg/dL/min was higher in the ITT (25.4%) compared to people with diabetes receiving subcutaneous insulin therapy (10.0%, *p* < 0.01) and people with insulinoma (2.6%, *p* < 0.01).

A significantly higher MARD was observed in people with a RoC <−1.0 mg/dL/min (MARD: 62.9) and a RoC −1 to <−0.5 mg/dL/min (MARD: 53.1), as compared to people with a RoC of ≥0.0–0.5 mg/dL/min (MARD: 35.8, *p* < 0.01, respectively) and a RoC of −0.5 to <0.0 mg/dL/min (MARD: 38.46, *p* = 0.02 and *p* = 0.08). Conversely, the proportion of values outside the %20/20 increased significantly from 16.2% at a RoC of ≥0.0 to 0.5 mg/dL/min and 19.3% at a RoC of −0.5 to <0.0 mg/dL/min to a proportion of 36.7% at a ROC of −1 to <−0.5 mg/dL/min (*p* < 0.1 and *p* 0.02) and 41.7% at a RoC of <−1.0 mg/dL/min (*p* < 0.01).

### 4.4 Special feature ITT

**FIGURE 3:**
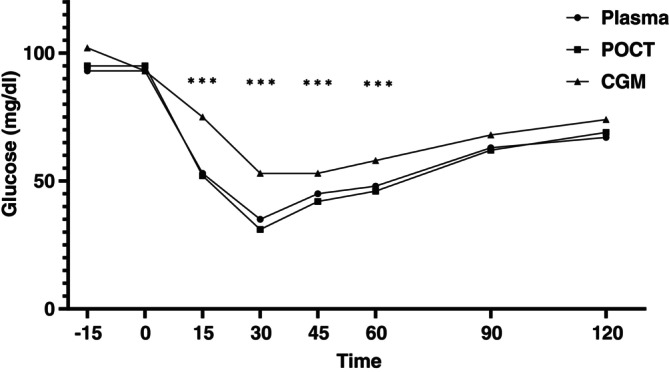
Glucose course during ITT according to measurement technique. Plasma, plasma glucose value; POCT, point of care glucose value; CGM, continuous glucose monitoring glucose value.***p < 0.01 for both comparison of CGM glucose versus plasma glucose and CGM glucose versus point of care glucose.

Regarding the possibility of comparing the course of Plasma‐G, POC‐G, and CGM‐G in ITT, the following was revealed: 15, 45 and 60 min after the insulin bolus injection, Plasma‐G and POC‐G were significantly lower than CGM‐G (*p* < 0.01 for each time point and for each comparison: Plasma‐G vs. CGM‐G, POC‐G vs. CGM‐G). However, at 90 and 120 min, glucose values between plasma, POC and CGM did not differ (Figure 3).

The accuracy and agreement of CGM‐G in reference to Plasma‐G were comparable to the CGM‐G versus POC‐G analysis listed above (Tables 1 and 2).

Furthermore, a comparison was made between the accuracy analyses based on CGM‐G values determined simultaneously with Plasma‐ and POC‐G and CGM‐G values collected 15 min later (i.e., CGM‐G at 30 min in reference to Plasma‐ and POC‐G at 15 min). The results showed no significant difference: overall MARD within the hypoglycaemic range was 52.1% (*p* 0.37) for CGM‐G in reference to POC‐G and 38.9% (*p* 0.34) for CGM‐G in reference to Plasma‐G. Percentage of values within the %20/20 was 69.1% (*p* 0.31) for CGM‐G in reference to POC‐G and 76.7% (*p* 0.29) for CGM‐G in reference to Plasma‐G. Again MARD was lower and percentage of values within the %20/20 higher within level 1 (MARD: CGM‐/POC‐G: 16.1%, CGM‐/Plasma‐G 18.5%, %20/20: CGM‐/POC‐G: 88.1%, CGM‐/Plasma‐G 80.5%) compared to level 2 hypoglycaemia (MARD: CGM‐/POC‐G: 62.1%, CGM‐/Plasma‐G 47.5%, %20/20: CGM‐/POC‐G: 62.1%, CGM‐/Plasma‐G 70.5%).

## 5 DISCUSSION

In this single‐centre, prospective study, we analysed the accuracy and reliability of a CGM in three different hypoglycaemia scenarios with different insulin administration routes. Our results showed that CGM accuracy varied significantly depending on the clinical scenario. The highest accuracy was observed in people with diabetes receiving subcutaneous insulin therapy, followed by moderate accuracy in people with insulinoma and impaired accuracy in people with ITT.

Data on the accuracy of CGM within the hypoglycaemic range is still sparse. For comparison, Wright et al. described a MARD of 7.6% in the hypoglycaemic range using the FreeStyle Libre 1/2 sensor in 77 people on subcutaneous insulin therapy. However, the MARD in their study was based on only five paired CGM/POC glucose values.^26^ Consequently, the external validity of their MARD within the hypoglycaemic range is severely limited. To contribute to the understanding of CGM accuracy within the hypoglycaemic range, our study included 464 CGM/POC‐G pairs distributed across this range in different scenarios. We demonstrated that CGM accuracy may vary in different hypoglycaemic scenarios, with MARD decreasing from 50.8% in people with ITT to 13.9% in people with diabetes receiving subcutaneous insulin therapy. While the former MARD may well lead to a misdiagnosis of hypoglycaemia, the latter MARD is acceptable, as it does not result in any deviation between CGM‐ and POC‐G >10.0 mg/dL in absolute terms. Furthermore, our MARD of 13.9% is consistent with a recent meta‐analysis, which showed a MARD of 13.6% for people with type 1 diabetes during physical exercise, resulting in an increased glucose RoC.^27^ In addition, the MARD is close to 10.0%, which is the recommended MARD as a basis for decisions regarding insulin dosing.^28^ However, it has been demonstrated that even sensors with MARDs >10.0% can offer a reliable approach to glycaemic management, and consequently, international guidelines explicitly endorse CGM use for management of insulin therapy.^29^, ^30^ Given the absence of CGM studies specifically addressing hypoglycaemic scenarios, the relevance of our findings is emphasised when considering that MARD values in very low glucose ranges can appear disproportionately high due to the calculation method. To illustrate, a MARD of 20.0% would only result in an absolute deviation of ±8.0 mg/dL for a blood glucose level of 40 mg/dL. In line with this, the DST error grid analysis of the three scenarios revealed that the proportion of CGM‐G values exceeding a minimal risk of failure in detecting hypoglycaemia was negligible from a clinical perspective for people with diabetes receiving subcutaneous insulin therapy and people with insulinoma. Nevertheless, a proportion of CGM‐G values that is clinically relevant and associated with a moderate risk of failure in detecting hypoglycaemia was evident in people with ITT.^24^

One possible explanation for the aforementioned stepwise decrease in CGM in our three hypoglycaemia scenarios is that subcutaneous insulin has a slower onset of action than intravenous insulin. Consequently, the intravenous application of insulin leads to a more rapid decrease in blood glucose values, which can be assessed by Plasma‐G and POC‐G, but not by CGM‐G, since CGM measures glucose in the subcutaneous tissue. Changes in blood sugar can be expected about 15–30 min later.^31^, ^32^, ^33^ Our presumption of impaired CGM accuracy during periods of rapid blood glucose decrease is supported by our observations that CGM accuracy significantly decreased with a higher glucose RoC, particularly when the RoC exceeded −1 mg/dL/min. This is also the range in which a significantly higher number of glucose pairs were located in the ITT than people with diabetes receiving subcutaneous insulin therapy and people with insulinoma. The correlation between increasing glucose RoC and decreasing CGM accuracy has been demonstrated in previous studies.^34^, ^35^ However, no studies have yet investigated this explicitly for the hypoglycaemic range. The issue of RoC in the hypoglycaemic range is further emphasised by our illustration of glucose curves depending on the measurement method selected in the ITT. Fifteen minutes after the insulin injection, a rapid decrease in Plasma‐ and POC‐G levels was observed, followed by a further decrease after 30 min and a slow increase from 30 to 120 min. In contrast, CGM‐G readings from 15 to 60 min were consistently higher and only levelled off after 60 min. Furthermore, even when comparing POC‐G and Plasma‐G with CGM‐G values obtained 15 min later, no improvement in CGM accuracy was demonstrated. Additionally, in the ITT and insulinoma scenarios, we revealed that level 2 hypoglycaemia was associated with impaired CGM accuracy compared to level 1 hypoglycaemia.

BAA of our CGM/POC‐G pairs revealed the highest estimated bias of −15.2 mg/dL in people with ITT compared to the lowest estimated bias of −3.3 mg/dL in people with diabetes receiving subcutaneous insulin. Consequently, it can be concluded that the CGM system examined tends to systematically overestimate glucose levels in the hypoglycaemic range.^25^ Nevertheless, again it is imperative to contextualise these biases within the clinical setting. Within the context of diabetes and insulinoma (mean bias −8.1 mg/dL), this systematic difference when compared to the gold standard POC‐G does not appear to be of clinical significance. However, the 95% limits of agreement for people with diabetes receiving subcutaneous insulin therapy and people with insulinoma indicate a considerably broad variability of CGM accuracy when focusing on the hypoglycaemic range.^36^ A notable limitation of the latter observation may be that it is an expression of too few value pairs, thereby hindering the generalisability of our observation.^36^ However, it can be concluded that, to ensure patient safety, a countermeasure using POC‐G is necessary when CGM indicates a glucose value <70 mg/dL. Furthermore, in people with ITT, where achieving a glucose value <40 mg/dL is imperative for the test's validity, the mean bias of −15.2 and the broad 95% limits of agreement indicating such a relevant inaccuracy that CGM use for glucose monitoring during the ITT should be refrained from.

## 6 LIMITATIONS AND STRENGTHS

We obtained our CGM/POC‐G pairs in people with insulinoma and diabetes receiving subcutaneous insulin therapy nearly simultaneously. Since glucose measurements were taken from different body compartments, changes in blood glucose levels will not be reflected in the subcutaneous tissue for 15 min.^33^ However, hypoglycaemia is an acute, life‐threatening condition that requires immediate detection and treatment. Furthermore, POC‐G rather than laboratory analysis of venous plasma glucose concentrations (Lab‐G) was used as the reference measurement to assess the accuracy of CGM in people with insulinoma and those with diabetes receiving subcutaneous insulin therapy. Studies comparing CGM‐G with POC‐G and Lab‐G have reported comparable MARD values for CGM‐G and Lab‐G, as well as CGM‐G and POC‐G. This was also demonstrated in our subgroup of people with ITT.^37^ Moreover, POC‐G measurement is the standard method for glucose monitoring in clinical practice guiding insulin treatment. Thus, our approach offers direct comparison of CGM with a real‐world comparator.^38^, ^39^ Another limitation is the external validity of the present study, since variations in CGM accuracy have been identified depending on the study design and the sensor investigated.^40^ This is primarily attributed to the absence of standardisation in the evaluation of CGM performance.^41^ Consequently, our results cannot be simply transferred to CGM systems of other manufacturers.

Our study's strength lies in its use of a well‐defined cohort with various causes and dynamics of hypoglycaemia. We obtained a large number of CGM/POC‐G data pairs within a wide hypoglycaemic range, and we performed a structured analysis of paired CGM‐ and POC‐G values. Furthermore, the ITT provides a unique opportunity to visualise the exact courses of plasma, POC, and CGM glucose curves side by side to draw conclusions about how blood glucose fluctuation dynamics influence CGM accuracy.

## 7 CONCLUSIONS

This study showed that CGM accuracy in the hypoglycaemic range is acceptable for people with diabetes receiving subcutaneous insulin therapy and people with insulinoma. However, in the case of ITT, CGM measurements demonstrate a tendency to lag behind. Therefore, CGM accuracy appears inaccurate for glucose monitoring during ITT.

## AUTHOR CONTRIBUTIONS

**Lukas van Baal:** Formal analysis, Investigation, Data Curation, Writing – Original Draft, Visualization. **Lorenz Raess:** Investigation, Data Curation. **Annie Mathew:** Writing – Review & Editing. **Harald Lahner:** Writing – Review & Editing. **Nicole Unger:** Resources, Writing – Review & Editing. **Dagmar Fuhrer:** Resources, Writing – Original Draft, Supervision, Project administration.

## CONFLICT OF INTEREST STATEMENT

Lukas van Baal, Lorenz Raess, Annie Mathew, Harald Lahner, Nicole Unger and Dagmar Fuhrer declare no conflict of interests.

## ETHICS STATEMENT

The study was approved by the ethics committee of the University of Duisburg‐Essen and was performed in accordance with the Declaration of Helsinki (approval number 20‐9333‐BO).

## Supporting information


**Data S1:** Supporting information.

## Data Availability

The data that support the findings of this study are available from the corresponding author upon reasonable request.
