# Supplementary material for: Accuracy and reliability of a continuous glucose monitoring system with a focus on hypoglycaemia
Source: Diabetes Obes Metab. 2025 Dec 25;28(3):2166–74. doi: 10.1111/dom.70408 (PMC12890745; doi:10.1111/dom.70408)
Supplement: Supplementary file 1 — Data S1: Supporting information. [file DOM-28-2166-s001.docx]

**Table 1: General characteristics of the study population. Values are given as mean+standard deviation or percentage affected**

|  | **Overall** | **ITT** | **Insulinoma** | **Subcutaneous insulin therapy** |
| --- | --- | --- | --- | --- |
| n | 92 | 63 | 16 | 13 |
| Age, years | 50.2±15.6 | 49.3±15.9 | 44.7±15.0 | 57.9±14.3 |
| Female, n (%) | 52(56.5) | 41 (65.1) | 54 (49.5) |  |
| BMI, kg/m^2^ | 27.7±8.3 | 28.3±5.4 | 27.1±7.3 | 27.5±9.2 |
| HbA1c, % (mmol/l) | 5.6±1.8 (85.0±9.0) | 5.8±0.5 (91.0±9.0) | 4.9±2.6 (79.0±9.0) | 6.7±1.7 |
| Hemoglobin, g/dl | 12.3±2.4 | 12.6±2.5 | 12.4±2.4 | 12.0±2.1 |
| Mild anemia hemoglobin (10-12 g/dl), n (%) | 10 (10.9) | 4 (6.4) | 4 (25.0) | 2 (15.4) |
| Moderate-severe anemia hemoglobin (<10 g/dl), n (%) | 2 (2.2) | 1(2.4) | 1 (6.3) | 0 (0.0) |
| Glomerular filtration rate (ml/min/1,73 m^2^) | 73.6±17.1 | 75.3±16.2 | 72.5±18.1 | 67.7±18.2 |
| Chronic kidney disease, n (%) | 8 (8.7) | 3 (4.8) | 2 (12.5) | 3 (23.1) |
| CKD G1 | 0 (0.0) | 0 (0.0) | 0 (0.0) | 0 (0.0) |
| CKD G2 | 4 (4.4) | 2 (3.2) | 1 (6.3) | 1 (7.7) |
| CKD G3a | 3 (3.3) | 1 (11.9) | 1 (6.3) | 1 (7.7) |
| CKD G3b | 1 (1.1) | 0 (0.0) | 0 (0.0) | 1 (7.7) |
| CKD G4 | 0 (0.0) | 0 (0.0) | 0 (0.0) | 0 (0.0) |
| CKD G5 | 0 (0.0) | 0 (0.0) | 0 (0.0) | 0 (0.0) |
| Total bilirubin, mg/dl | 0.6±0.6 | 0.5±0.8 | 0.7±0.4 | 0.8±0.2 |
| Acetylsalicylic acid, n (%) | 12 (13.0) | 7 (11.1) | 2 (12.5) | 3 (23.1) |
| Ascorbic acid, n (%) | 0 (0) | 0 (0) | 0 (0) | 1 |
| Acetaminophen, n (%) | 2 (2.2) | 1 (2.4) | 1 (1.0) | 0 (0.0) |
| Tetracycline, n (%) | 0 (0.0) | 0 (0.0) | 0 (0.0) | 0 (0.0) |
| Hydroxyurea | 0 (0.0) | 0 (0.0) | 0 (0.0) | 0 (0.0) |

CGM, continuous glucose monitoring; n, number; BMI, body mass index; g, gram; dl, decilitres; ml, millilitres; min, minute, CKD, chronic kidney disease; mg, milligram
